# The effect of Liv-52 on liver ischemia reperfusion damage in rats

**DOI:** 10.1186/s40360-019-0380-0

**Published:** 2020-01-03

**Authors:** Orhan Cimen, Hüseyin Eken, Ferda Keskin Cimen, Arif Burak Cekic, Nezahat Kurt, Asli Ozbek Bilgin, Bahadir Suleyman, Halis Suleyman, Renad Mammadov, Kamil Pehlivanoglu, Eray Kurnaz

**Affiliations:** 1 Department of General Surgery, Faculty of Medicine, Erzincan Binali Yildirim University, 24100 Erzincan, Turkey; 2 Department of Pathology, Faculty of Medicine, Erzincan Binali Yildirim University, 24100 Erzincan, Turkey; 3 Department of General Surgery, Faculty of Medicine, Karadeniz Technical University, 61000 Trabzon, Turkey; 4 Department of Biochemistry, Faculty of Medicine, Erzincan Binali Yildirim University, 24100 Erzincan, Turkey; 5 Department of Pharmacology, Faculty of Medicine, Erzincan Binali Yildirim University, 24100 Erzincan, Turkey

**Keywords:** Ischemia reperfusion, Injury, Liver, Liv-52, Rat

## Abstract

**Background:**

Liver ischemia reperfusion (I/R) damage which is frequently seen in clinical hepatobiliary surgeries has no effective treatment for it. Liv-52, known to have hepatoprotective effects, is a natural antioxidant drug licensed by the Ministry of Health of India. The aim of our study is to investigate the effect of Liv-52 on liver damage induced by I/R in rats.

**Methods:**

Albino Wistar male rats were divided into three groups; liver I/R (IR), 20 mg/kg Liv-52 + liver ischemia reperfusion (LIR) and sham operation applied to control group (HG). Liv-52 was administered to the LIR group (*n* = 6) 1 h prior to I/R application and distilled water was given orally to IR (*n* = 6) and HG (*n* = 6) groups as a solvent. Ischemia was determined as 1 h, and reperfusion was identified as 6 h in animals.

**Results:**

Increased levels of alanine aminotransferase, aspartate aminotransferase and lactate dehydrogenase, malondialdehyde, myeloperoxidase, and decreased levels of superoxide dismutase, and glutathione related enzymes caused by I/R application have been converged to healthy group level with Liv-52 treatment and the damage in liver tissue has been improved histopathologically.

**Conclusions:**

Liv-52 may be beneficial for preventing liver I/R damage in pre-surgery application.

## Background

Hepatic portal occlusion operation or Pringle maneuver interrupting portal vein and hepatic artery entries are temporarily performed to control bleeding control during operations such as liver resection, transplantation and hepatobiliary surgery [1]. This method is widely used since it is clinically simple, practical and effective. However, this methodology can lead to a significant risk of liver damage due to ischemia reperfusion (I/R) [2]. The formation of excessive free radicals is responsible for the reperfusion damage and for the molecular oxygen presented in great amounts with arterial blood to the ischemic tissue [3]. Free oxygen radicals cause cell membrane lipids to oxidize and allow toxic products such as malondialdehyde (MDA) from lipids [4]. The information obtained from the literature suggests that I/R damage is a pathological process that begins with the asphyxiation of the tissues and continues with the production of free oxygen radicals [5]. Therefore, a current shared hypothesis suggests antioxidant drugs may be useful for preventing I/R damage. In this study, we will examine the effects of Liv-52 against I/R liver damage which is licensed as an ayurvedic medicine by the AYUSH department, a drug regulatory authority of the Indian Ministry of Health. Each tablet of Liv-52 contains medicinal plants extracts at specified doses: 65 mg of *Capparis spinosa*, 65 mg of *Cichorium intybus*, 33 mg of Mandur Bhasma, 32 mg of *Solanum nigrum*, 32 mg of Terminalia Arjuna, 16 mg of *Cassia occidentalis*, 16 mg of *Achillea millefolium* and 16 mg of *Tamarix gallica* [6]. It has been reported that Liv-52 protects liver against toxicity of ethanol by preventing the increase of lipid peroxidation and the reduction of antioxidants in rat liver tissue [7].

The information obtained suggests that Liv-52 may be useful during I/R treatment or may protect the liver tissue from I/R damage. There was no information on the protective effect of Liv-52 against liver I/R damage in literature review. For this reason, the aim of our study is to examine the effect of Liv-52 on the liver damage induced by I/R in rats biochemically and histopathologically.

## Methodology

### Animals

The experimental animals have been received from Atatürk University Medical Experimental Application and Research Center. There were totally 18 experimental animals used in this study and they were all male albino Wistar rats. Their weights varied between 250 and 270 g (8–10 months). The rats have been kept until the surgery in groups under proper temperature and conditions. The temperature was adjusted as room temperature (22 °C ± 1 °C). They were maintained in a 12:12-h light–dark cycle. The study has been carried out according to the National Guidelines for the Use and Care of Laboratory Animals. Moreover the study received the approval from the local animal ethics committee in Ataturk University, Erzurum, Turkey (Ethics Committee Number: 77040475–000-E.1700216877, Dated:03.08.2017).

### Chemicals

Of the chemical substances used for the experiments, thiopental sodium was received from IE.

Ulagay, Istanbul, Turkey. Liv-52 was received from Himalaya Drug, Shankar Nagar, Nagpur, Maharashtra, India.

### Experimental groups

Experimental animals have been categorized in three different groups, with 6 rats in each group and treated as follows: liver ischemia/reperfusion (IR), 20 mg/kg Liv-52+ liver ischemia reperfusion (LIR) and sham operation applied to the healthy group (HG).

### Experimental procedure

The surgical interventions on rats were carried out under sterile conditions. Anesthesia was performed by administering 25 mg/kg of intraperitoneal (ip) thiopental sodium and xylazine by inhalation at appropriate intervals. One hour before thiopental sodium anesthesia, Liv-52 was given to the LIR animal group orally by a catheter at a dose of 20 mg/kg and volume of 1 cc as given in previous studies [8]. Also, 100 mg/kg Liv-52 studied for isoniazid and rifampicin induced hepatotoxicity [9]. 20 mg for 1000 mg rat, each rat weighs between 250 and 270 mg. One tablet weighing 320 mg dissolved in 64 cc distilled water and 1 cc given by gavage orally. 1 cc has 5 mg Liv52(20 mg/kg) and also contains as one tablet (weighing 320 mg) of Liv52 contains; *Capparis spinosa* %20,3125, *Cichorium intybus* %20,3125, Mandur Bhasma %10,3125, *Solanum nigrum* %10, Terminalia Arjuna %10, *Cassia occidentalis* %5, *Achillea millefolium* %5 and *Tamarix gallica* % 5. Distilled water as solvent was administered to the IR and HG rat groups with the same method. After the injection of thiopental sodium, the rats were kept for the appropriate surgery period. Surgical intervention was applied after the period when the animals were motionless in supine position was considered to be appropriate. During this process, all the rats were brought to supine position and laparotomy was performed by the 3,5–4 cm long vertical dissection of the abdomen’s anterior portion. Later, one hour for ischemia was performed by placing clamps on the hepatic artery, portal vein and bile duct in order to create total hepatic ischemia (excluding the HG group). And after ischemia period 6 h of reperfusion was provided. At the end of reperfusion period, one blood sample for each rat (each sample replicated three times for biochemical analyzes and averaged) was taken from the tail veins of the animals for the measurement of Alanine aminotransferase (ALT), Aspartate aminotransferase (AST) and Lactate dehydrogenase (LDH) activities. Later, rat groups were killed with high dosage of anesthesia (50 mg/kg i.p. thiopental sodium IE Ulagay-Türkiye) and their liver tissues were removed. Oxidant/antioxidant parameters were determined from the removed tissues and the tissues were examined histopathologically.

### Biochemical measurements

#### Serum alanine aminotransferase, aspartate aminotransferase and lactate dehydrogenase measurements

Venous blood samples collected into tubes without anticoagulant. Serum was separated by centrifugation after clotting and stored at − 80 °C until assay. Serum AST and ALT activities as liver function tests, and LDH activity as a marker of tissue injury, were measured spectrophotometrically on a Cobas 8000 (Roche) autoanalyser using commercially available kits (Roche Diagnostics, GmBH, Mannheim, Germany).

#### Sample preparation for analyses of biochemical parameters

20 mg of liver tissue weighed for each liver and the samples homogenized in ice with 2-mL buffers (consisting of 0.5% hexadecyltrimethylammonium bromide) pH 6 potassium phosphate buffer for myeloperoxidase analyze, consisting of 1.15% potassium chloride solution for thiobarbituric acid reactions (TBARS) analysis and pH 7.5 phosphate buffer for the superoxide dismutase, total glutathione analysis. Then, they centrifuged at 4 °C, 10.000×g for 15 min. The supernatant part used as the analysis sample. And each sample replicated three times for biochemical analyzes and averaged. The protein concentration of the supernatant measured with the method described by Bradford [10].

#### Malondialdehyde analysis

The method of Ohkawa H et al. used for MDA measurement [11]. This method was based on the spectrophotometric measurement (at a wavelength of 532 nm) of the absorbance of the pink colored complex created by thiobarbituric acid (TBA) and MDA at a high temperature (95 °C). The corpus mucosa was scraped, weighed, and homogenized in 10 ml of 100 g/L KCl. The homogenate (0.5 ml) was added to a solution containing 0.2 ml of 80 g/l sodium lauryl sulfate, 1.5 ml of 200 g/l acetic acid, 1.5 ml of 8 g/L 2-thiobarbiturate, and 0.3 ml distilled water. The mixture incubated at 98 °C for 1 h. Upon cooling, 5 ml of n-butanol:pyridine (15:l) added. The mixture vortexed for 1 min and centrifuged for 30 min at 1800×g. The absorbance of the supernatant measured at 532 nm. The standard curve obtained using 1,1,3,3- tetramethoxypropane (1.56–3.12 - 6.25 - 12.5 - 25 - 50 - 100 μM) .

#### Myeloperoxidase analysis

The activity of myeloperoxidase (MPO) in the total homogenate was measured according to the method of Wei and Frenkel with some modifications [12]. The sample was weighed and homogenized in 2 ml of 50 mmol/L phosphate buffer containing 0.5% hexadecyltrimethyl ammonium bromide (HDTMAB) and centrifuged at 1200×g for 60 min at 4 °C. The supernatant was used to determine MPO activity using 1.3 mL 4-aminoantipyrine-2% phenol (25 mM) solution. 25 mmol/L 4-aminoantipyrine–2% phenol solution and 1.5 mL of 0.0005% H_2_O_2_ were added and equilibrated for 3–4 min. After establishing the basal rate, a 0.2 mL sample suspension was added and quickly mixed. Increases in absorbance at 510 nm for 4 min at 0.1-min intervals were recorded. Absorbance was measured at 412 nm using a spectrophotometer.

#### Superoxide dismutase analysis

The method of Sun et al. used for the measurement of superoxide dismutase [13]. Xanthine is converted into uric acid using xanthine oxidase. When nitro blue tetrazolium (NBT) was mixed into the reaction, SOD reacted with NBT. The color of the formazan dye became purple. The weight measurement was carried out and the mixture was homogenized in 2 ml of 20 mmol/L phosphate buffer containing 10 mmol/L EDTA at pH 7.8. Then centrifugation was performed at 3600×g for 10 min. The supernatant was preferred as the sample of assay. The sample containing 2450 μL mixture (0.3 mmol/L xanthine, 0.6 mmol/L EDTA, 150 μmol/L NBT, 0.4 mol/L Na 2 CO 3, 1 g/l bovine serum albumin), 500 μL supernatant and 50 μL xanthine oxidase (167 U/l) was vortexed. Afterwards the incubation was carried out for 10 min. Formazan was formed as the result of the reaction. The measurement of absorbance was made on the formazan in purple color at 560 nm. If there would be more enzymes, then there would be a decrease in O2 − radical reacting with NBT.

#### Total glutathione analysis

The measurement of GSH in the total homogenate was carried out by using the method prepared by Sedlak and Lindsay with few changes [14]. Then the weight of the sample was measured and it was homogenized in 2 mL of 50 mmol/L Tris–HCl buffer with 20 mmol/L EDTA and 0.2 mmol/L sucrose at pH 7.5. The mixture was precipitated with 0.1 mL of 25% trichloroacetic acid, Afterwards, the precipitate was centrifuged at 1800×g for 40 min at 4 °C. Supernatant was preferred in order to identify the level of GSH. 1500 μL of measurement buffer (200 mmol/L Tris–HCl buffer containing 0.2 mmol/L EDTA at pH 7.5), 500 μL supernatant, 100 μL DTNB (10 mmol/L) and 7900 μL methanol have been mixed and then vortexed. The mixture was exposed to incubation for 30 min in 37 °C. 5.5-Dithiobis (2-nitrobenzoic acid) was used as a chromogen. The color was formed into yellow with sulfhydry groups. The measurement of absorbance has been carried out at 412 nm by means of a spectrophotometer (Beckman DU 500, USA). Finally, a standardized curve was found by means of decreased glutathione (0.5–1 - 2 - 4 - 8 - 16 - 32 μM).

#### Glutathione peroxidase analysis

GPO activity determined by the method of Lawrence and Burk [15]. After the KH2PO4, EDTA, GSH, B-NADPH, NaN3, and GR addition, mixture was incubated. As soon as H2O2 was added, and the absorbance was carried out every 15 s for 5 min, at 340 nm.

#### Glutathione reductase analysis

GSHRd activity determined spectrophotometrically by measuring the rate of NADPH oxidation at 340 nm according to Carlberg and Mannervik method [16]. After the NADPH and GSSG addition, the absorbance measured for 5 min by 30-min intervals at 340 nm spectrophotometric methods.

#### Glutathione S transferase activity

GST activity was determined by Habig and Jakoby [17]. Briefly, the enzyme’s activity was assayed spectrophotometrically at 340 nm in a 4-ml cuvette containing 0.1 M PBS (pH 6.5), 30 mM GSH, 30 mM 1-chloro-2,6-dinitrobenzene, and tissue homogenate.

### Histopathologic examination

Liver tissues of rats were fixed in 10% formalin solution for 24 h. All the tissues processed routinely, then 4 μm thick sections obtained from the paraffin blocks and stained with Hematoxylin&Eosin. All sections were examined under a light microscope (Olympus BX 52, Tokyo, Japan) by two pathologists who is aware of which treatment protocol used.

### Statistical analyses

The results obtained from the experiments are depicted as “mean ± standard error” (x ± SEM). Normality of the data was tested with Shapiro-Wilk test. All the parameters showed normal distribution. The significance level of the inter-group difference was identified using one-way ANOVA test. Then, Bonferroni post-hoc test was performed. All statistical analyses were performed using “IBM SPSS Statistics Version 22” program and *p* < 0.05 was considered significant.

## Results

### Biochemical results

#### Effect of Liv-52 supplementation and I/R on liver enzymes

**Fig. 1:**
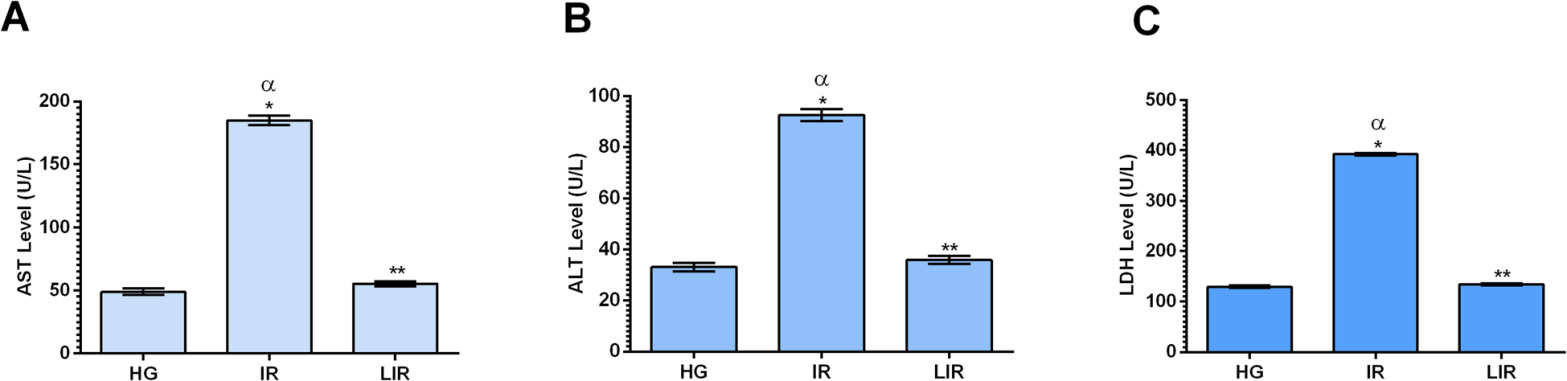
AST (**a**), ALT (**b**), LDH (**c**) levels of HG, IR and LIR groups. (*n* = 6), * = *P* < 0.001, ** = *P* > 0,05 in comparison to HG group

As showed in Fig. 1, the AST, ALT and LDH activities used in evaluating the liver functions were increased in I/R group compared to healthy group and there was a statistically significant difference (*p* < 0.001) between them. This increase due to I/R was suppressed by Liv-52 application and the difference between I/R group and Liv-52 group was statistically significant(*p* < 0.001). The difference between the healthy group and the Liv-52 group was not significant (*p* > 0.05) (Table 1).

**Table 1:** Biochemical results of the experimental groups

	HG (Mean ± Standard error)	IR (Mean ± Standard error)	LIR (Mean ± Standard error)
AST (IU/L)	48.83 ± 2.63	184.83 ± 3.72^a,b^	55,17 ± 2,09^c^
ALT (IU/L)	33.00 ± 1.73	92.50 ± 2.36^a,b^	35.83 ± 1.54^c^
LDH (IU/L)	129.33 ± 2.63	392.33 ± 2.63^a,b^	133.83 ± 1.70^c^
MDA (μmol/ g protein)	1.57 ± 0.15	4.60 ± 0.18^a,b^	1.97 ± 0.15^c^
MPO(U/g protein)	2.53 ± 0.17	5.93 ± 0.22^a,b^	2.75 ± 0.22^c^
SOD(U/g protein)	11.33 ± 0,80	3.35 ± 0.18^a,b^	9.13 ± 0.28^c^
tGSH (nmol/g protein)	25.67 ± 1.12	3.78 ± 0.20^a,b^	19.67 ± 1.12
GPO(U/g protein)	28.67 ± 0.88	4.73 ± 0.18^a,b^	23.67 ± 1.45
GSHRd(U/g protein)	17.33 ± 1.28	5.98 ± 0.17^a,b^	14.00 ± 0.97^c^
GST(U/g protein)	21.67 ± 0.88	7.30 ± 0.23^a,b^	18.50 ± 1.18^c^

*Abbreviations*: *HG* Healthy group, *IR* Liver ischemia/ reperfusion, *LIR* 20 mg/kg Liv-52+ liver ischemia reperfusion, *AST* Aspartate aminotransferase, *ALT* Alanine aminotransferase, *LDH* Lactate dehydrogenase, *MDA* Malondialdehyde, *MPO* Myeloperoxidase, *SOD* Superoxide dismutase, *GSH* Total glutathione, *GPO* Glutathione peroxidase, *GSHRd* Glutathione reductase, *GST* Glutathione S transferase. ^a^means, *p* < 0.001 compared to healthy group, ^b^means *p* < 0.001 compared to Liv-52+ IR group, ^c^means *p* > 0.05 compared to healthy group

#### Effect of Liv-52 supplementation on lipid peroxidation and antioxidant status in liver tissue

**Fig. 2:**
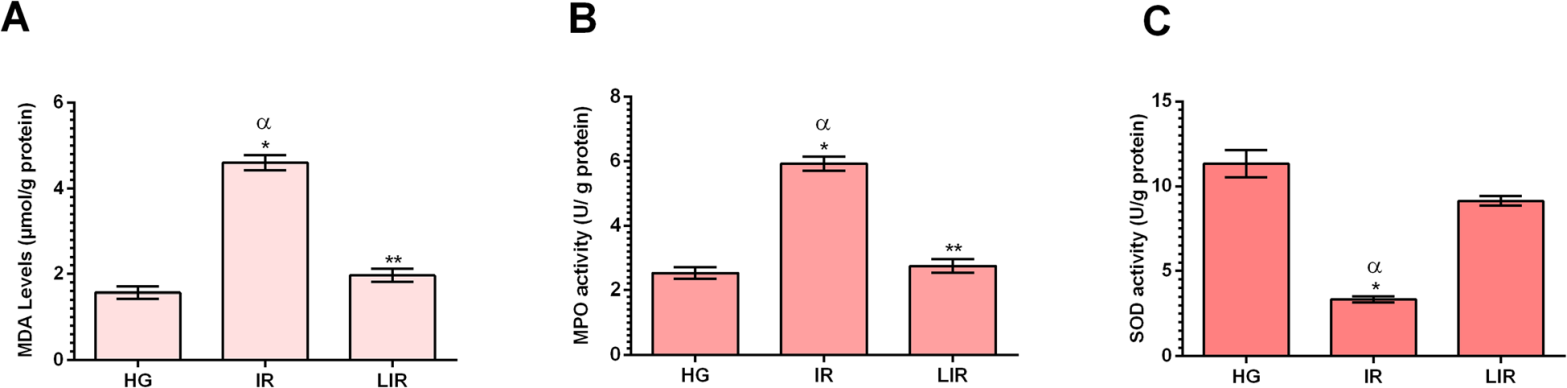
MDA levels (**a**), MPO activity (**b**), SOD activity (**c**) in the liver tissues of HG, IR and LIR groups. (*n* = 6), * = *P* < 0.001, ** = *P* > 0,05 in comparison to HG group

As showed in Fig. 2a, the liver MDA level increased with I/R application in comparison to the healthy group (*p* < 0.001). In the group treated with Liv-52, this rise due to I/R was suppressed and the level was decreased. The difference between I/R and Liv-52 was significant (*p* < 0.001). There was no significant difference between HG and Liv-52 group (*p* > 0.05). MPO activity was increased due to I/R application in comparison to the HG (*p* < 0.001). In the Liv-52 group, MPO level almost decreased to the level of the HG (*p* > 0.05) and the difference between I/R and Liv-52 was significant (*p* < 0.001) (Fig. 2b). SOD activity decreased with I/R application according to HG (*p* < 0.001). Also the difference between I/R and Liv-52 was significant (*p* < 0.001) and Liv-52 suppressed the decrease in SOD activity and elevated SOD activity to nearly activity of the HG (Fig. 2c) (Table 1).

#### Effect of Liv-52 supplementation on tGSH level and activities of glutathione-dependent enzymes in liver tissue

**Fig. 3:**
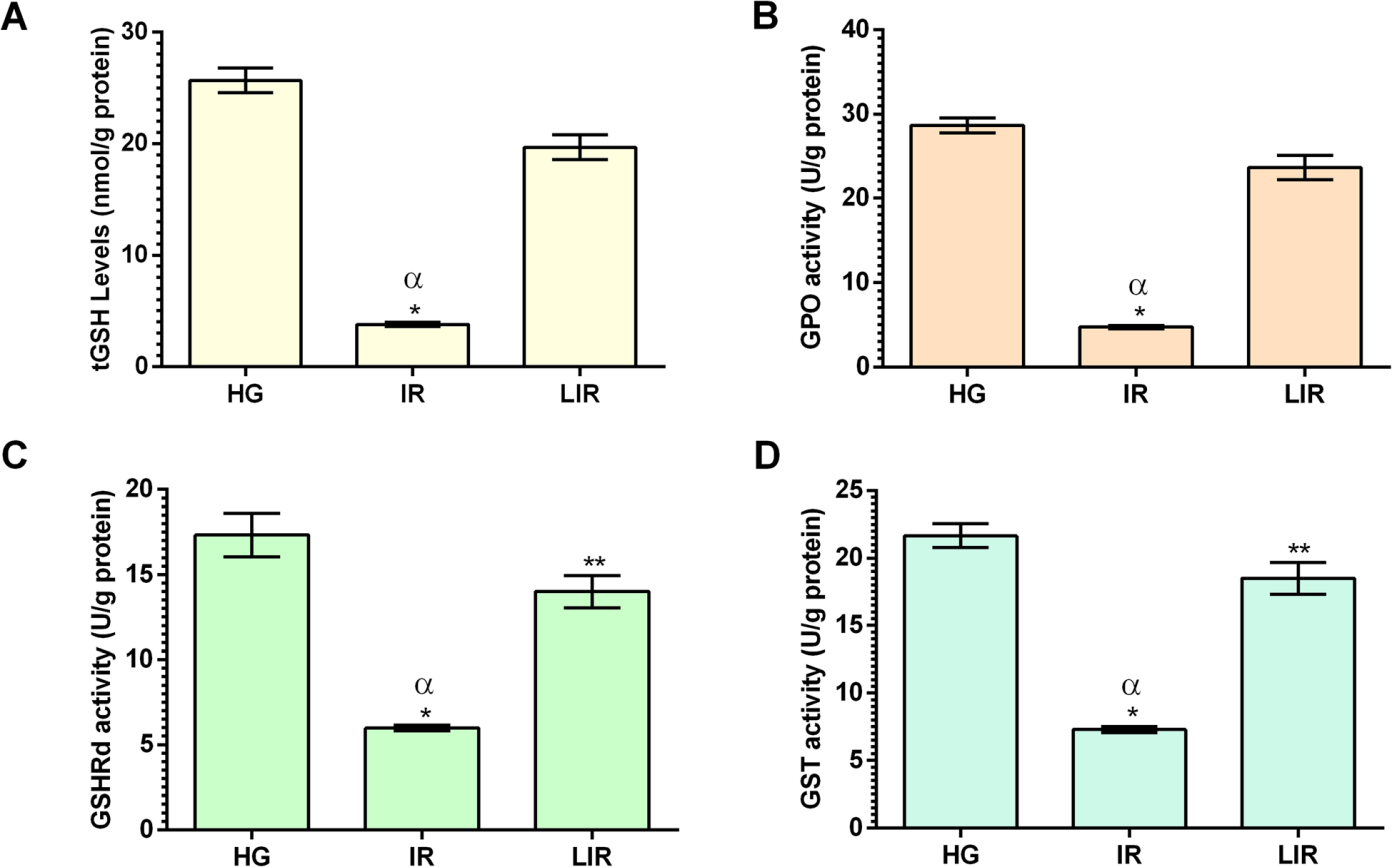
tGSH (**a**) level and activities of glutathione-dependent enzymes GPO (**b**), GSHRd (**c**), GST (**d**) in liver tissues of HG, IR and LIR groups. (*n* = 6), * = *P* < 0.001, ** = *P* > 0,05 in comparison to HG group

As shown in Fig. 3a, tGSH level decreased with I/R application compared to the HG(*p* < 0.001). Liv-52 increased the level of tGSH again. There was a significant difference between I/R group and the Liv-52 group (*p* < 0.001).

GPO activity was 28,67 ± 0,88 U/g protein in HG and I/R application caused a decrease(*p* < 0.001). This decline due to I/R was suppressed by the application of Liv-52 and increased again, there was a significant difference (*p* < 0.001) between these two groups (Fig. 3b).

As it can be seen in Fig. 3c, liver GSHRd activity decreased in the I/R group according to the HG (*p* < 0.001). This value increased by Liv-52 application, difference between Liv-52 and the HG was not significant (*p* > 0.05), but the difference between I/R group and Liv-52 group was significant (*p* < 0.001).

GST activity decreased with I/R application compared to the HG and there was a significant difference between these two groups (*p* < 0.001). GST activity increased with Liv-52 application, and the difference between Liv-52 group and the HG was not significant (*p* > 0.05) but there was a significant difference between I/R and Liv-52 groups (*p* < 0.001) (Fig. 3d) (Table 1).

### Histopathological results

**Fig. 4:**
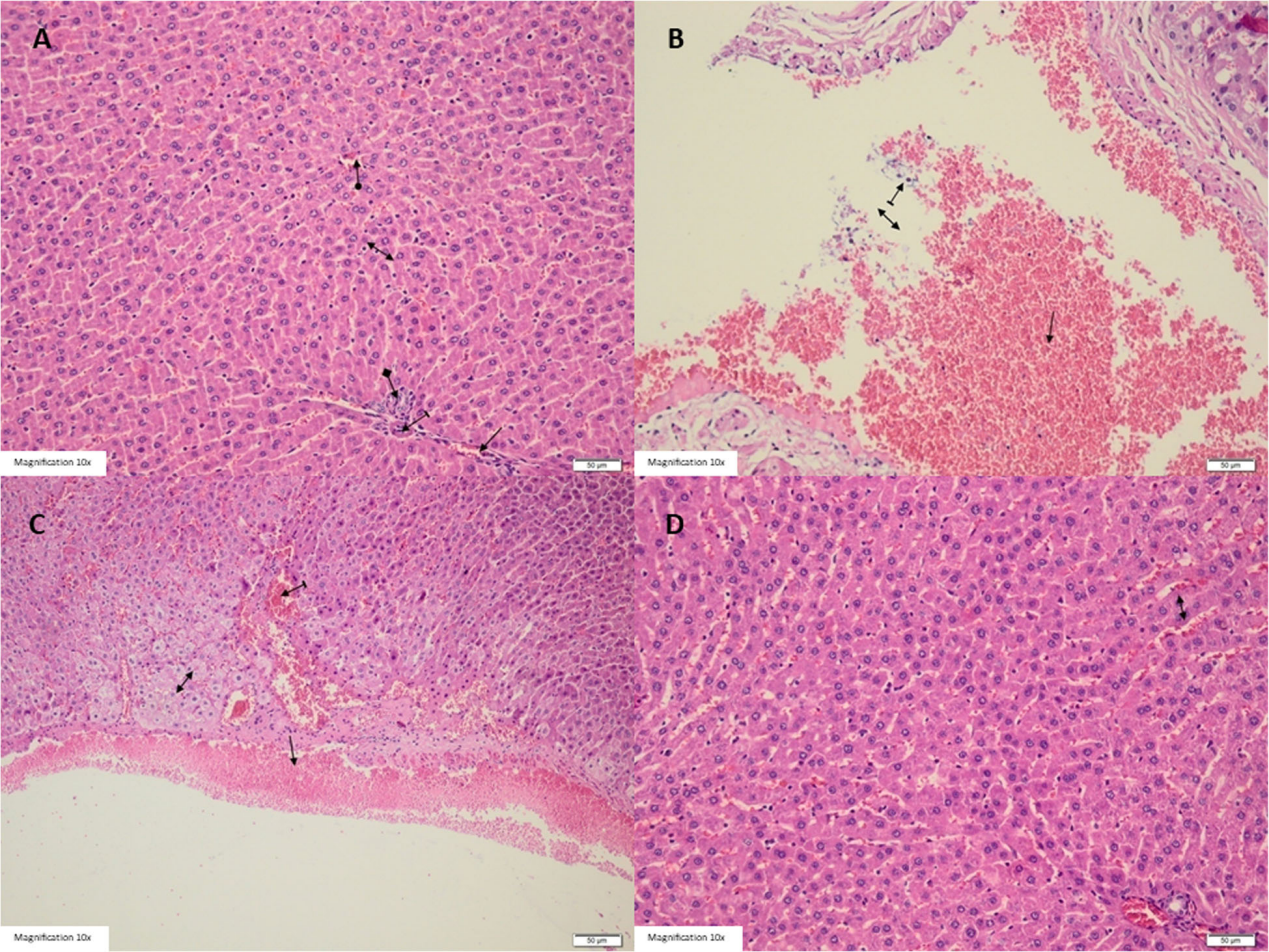
Histopathological findings of the liver tissues: **a** Optical microscopic view of HG group: Healthy liver portal vein (straight arrow), artery (dashed arrow), bile ductus (square arrow), central vein (circular arrow), liver parenchyma (two-way arrow). **b** Optical microscopic view of IR group: Hemorrhage (straight arrow), edema (two-way arrow) and polymorph nuclear leukocytes(dashed arrow) are observed in the ischemia reperfusion applied liver tissue (HEX200). **c** Optical microscopic view of IR group: Dilated congested blood vessels (straight arrow), dilate congested sinusoids (dashed arrow) and cells showing balloon degeneration (two way arrow) are observed in the ischemic reperfusion applied liver tissue (HEX 200) and (**d**). Optical microscopic view of LIR group: Near-normal liver tissue is observed in the group treated wofith Liv-52 except for a slight sinusoidal dilation and congestion of the liver tissue (HEX 200)

As shown in Fig. 4a, healthy group displayed normal liver parenchyma, portal vein, artery, bile ductus and central vein. Conversely, the liver tissue of the I/R applied group showed, common hemorrhage, edema, dilated congested blood vessels, dilated congested sinusoids, cells showing balloon degeneration and polymorphic leukocytes (Fig. 4b, c). Near-normal liver tissue was observed in the group treated with Liv-52 except for slight sinusoidal dilation and congestion in the liver tissue (Fig. 4d).

## Discussion

Hemorrhage control procedures performed during surgical operations on blood-rich liver causes I/R damage [18]. Recently, clinical studies carried out in the field of hepatic surgery have focused on how to reduce blood loss and I/R damage more safely [19]. Especially lately, the experimental application of antioxidants on liver I/R damage is also important for understanding the underlying mechanisms. In this study, the effect of the antioxidant features of Liv-52 against liver I/R damage have been examined and the results of the study showed biochemically and histopathologically that Liv-52 exerts a protective effect, preventing oxidative stress and apoptotic tissue damage formed by liver I/R damage.

I/R damage leads to the release of enzymes such as ALT which is a specific indicator for cytolysis and hepatic parenchymal damage and AST which is a nonspecific marker [20] . Studies have shown that LDH levels reflecting ALT, AST, and acute liver damages are significantly increased compared to normal group after I/R application [2]. Similarly, I/R significantly increased ALT, AST and LDH levels in our study. It is known that Liv-52, a herbal formulation rich in phenolic compounds, significantly reduces infections caused by biological agents and liver damage formed after chemical toxins in humans and experimental animals [21, 22]. There are studies in literature, recommending Liv-52 for liver protection against various hepatotoxins, per 2 or 3 times a day [23]. Also, Liv-52 has been reported to reduce liver enzymes to levels ​​close to the control group in liver damage that is formed by isoniazide and rifampicin [9]. Similarly, increase in liver enzymes due to I/R damage decreased to the level of the healthy group with the application of Liv-52 in our study.

It is also known that Liv-52 suppressed the increased levels of MDA, which is a marker of lipid peroxidation, and SOD, which clears intracellular free radicals, in tert-butyl hydroperoxide induced hepatotoxicity [22]. It has been suggested that liver ischemia affects mitochondrial energy synthesis and electron transport in the respiratory chain as a cause of hypoxia [24], thus produces a large number of free oxygen radicals that triggering cell damage through lipid peroxidation in biological membranes [25].

The studies carried out have shown that I/R application increases oxidative stress parameters such as MDA as well as MPO in the liver and decreases SOD activity [26, 27]. In our study, I/R application increased MDA and MPO levels and decreased SOD activity in accordance with the previous data. The Liv-52 application reversed this condition and showed hepatoprotective effect. There has been no study showing the effect of Liv-52 on MPO in the literature. Our study has indicated that Liv-52 alleviates the inflammatory reaction in the damaged liver tissue which I/R-induced by reducing the hepatic MPO activity.

As known, GSH being an effective antioxidant that protects cells from the damage of the free radicals formed by I/R is present at high concentrations in hepatocytes [28]. Deng et al. reported that the GSH value of the I/R group significantly decreased compared to the normal group and that the GSH value of the I/R + melatonin applied group significantly increased compared to the I/R group after reperfusion, at the 2nd, 4th and 8th hours [2]. In a toxicity study, it has been revealed that Cu^2+^ application reduced GSH in HepG2 cells by 86% and that Liv 52 application significantly increased GSH levels in toxic conditions induced by Cu^2+^ by 74%. In our study, the significantly decreased level of GSH compared to I/R and healthy groups significantly increased with the application of Liv-52 in accordance with the literature.

Reductions in the activities of GPO, GSHRd and GST being glutathione-associated antioxidant enzymes due to liver I/R damage have been previously reported [29, 30]. GPO, GSHRd, and GST activities in the I/R damaged liver tissue significantly decreased compared to the healthy group in our study. The Liv-52 application suppressed this decline and brought the value to a level close to level of healthy group. Previous studies showed that Liv-52 significantly increased the levels of serum GPO, reduced glutathione and GST which had been reduced in the paracetamol-induced liver toxicity group in the literature [31]. This information has also supported the results of our study.

The effects of Liv-52 on the liver damage induced by I/R were also examined histopathologically in our study. It’s shown that I/R application causes degeneration in hepatocyte, vein and intercellular edema and congested sinusoidal damage in the rat liver [32]. It has also been reported that, by the application of Liv-52, hepatocytes become normal histopathologically in the cadmium-induced hepatic toxicity and Liv-52 reverses cadmium-induced hepatic damage [33]. In our study, findings such as hemorrhage, edema, dilated congested blood vessels caused by I/R were reduced with the application of Liv-52 and near-normal liver tissue was observed. It can be seen with this information that Liv-52, which is known to have hepatoprotective effects previously, also has protective effects on liver I/R damage.

## Conclusion

I/R application leads to liver dysfunction and oxidative liver damage. I/R application changed the oxidant antioxidant balance in favor of oxidants. Liv-52 prevented this disequilibrium as well as the I/R-associated hepatic dysfunction. Based on the literature information and our experimental results, we can confirm that Liv-52 has a therapeutic effect that reduces hepatic damage induced by I/R.

## Data Availability

There is no data other than the data given in the article.

## Abbreviations

ALT: Alanine aminotransferase
AST: Aspartate aminotransferase
GSHR: Glutatione reductase
GSHRd: Glutathione reductase
GST: Glutathione s Transferase
HG: Control group
I/R: Ischemia reperfusion
LDH: Lactate dehydrogenase
LIR: 20 mg/kg Liv-52 + liver ischemia reperfusion
MDA: Malondialdehyde
MPO: Myeloperoxidase
SOD: Superoxide dismutase
tGSH: Total glutathione
